# Development of an algorithm for assessing fall risk in a Japanese inpatient population

**DOI:** 10.1038/s41598-021-97483-1

**Published:** 2021-09-09

**Authors:** Tomoko Nakanishi, Tokunori Ikeda, Taishi Nakamura, Yoshinori Yamanouchi, Akira Chikamoto, Koichiro Usuku

**Affiliations:** 1 Department of Medical Information Science, Graduate School of Medical Sciences, Kumamoto University, 1-1-1 Honjo, Chuou-ku Kumamoto, 860-8556 Japan; 2 Department of Nursing, Kumamoto University Hospital, Kumamoto, Japan; 3 Department of Medical Information Sciences and Administration Planning, Kumamoto University Hospital, Kumamoto, Japan; 4 Laboratory of Clinical Pharmacology and Therapeutics, Faculty of Pharmaceutical Sciences, Sojo University, 4-22-1 Ikeda, Nishi-ku Kumamoto, 860-0082 Japan; 5 Department of Medical Quality and Safety Management, Kumamoto University Hospital, Kumamoto, Japan

**Keywords:** Disease prevention, Geriatrics, Public health

## Abstract

Falling is a representative incident in hospitalization and can cause serious complications. In this study, we constructed an algorithm that nurses can use to easily recognize essential fall risk factors and appropriately perform an assessment. A total of 56,911 inpatients (non-fall, 56,673; fall; 238) hospitalized between October 2017 and September 2018 were used for the training dataset. Correlation coefficients, multivariable logistic regression analysis, and decision tree analysis were performed using 36 fall risk factors identified from inpatients. An algorithm was generated combining nine essential fall risk factors (delirium, fall history, use of a walking aid, stagger, impaired judgment/comprehension, muscle weakness of the lower limbs, night urination, use of sleeping drug, and presence of infusion route/tube). Moreover, fall risk level was conveniently classified into four groups (extra-high, high, moderate, and low) according to the priority of fall risk. Finally, we confirmed the reliability of the algorithm using a validation dataset that comprised 57,929 inpatients (non-fall, 57,695; fall, 234) hospitalized between October 2018 and September 2019. Using the newly created algorithm, clinical staff including nurses may be able to appropriately evaluate fall risk level and provide preventive interventions for individual inpatients.

## Introduction

Falls in elderly people often occur and are one of the major factors depriving them of their independence^1^. Falls are classified as E880–E888 in International Classification of Disease-9 (ICD-9), and as W00–W19 in ICD-10, which include a wide range of falls including those on the same level, upper level, and other unspecified falls^2^. The World Health Organization (WHO) has defined falls as “inadvertently coming to rest on the ground, floor or other lower level, excluding intentional change in position to rest in furniture, wall or other objects”^3^.

The incidence of falls increases with aging. In Japan, the aging rate has increased to 28.4% and the average life expectancy has reached 87.5 years old for women and 81.4 years old for men^4^. Accordingly, the incidence rate of falls in local elderly people is approximately 20% per year in Japan^5,6^. Falls in older individuals are most often due to multiple causes such as muscle weakness, age-related declines in balance, gait stability, and cardiovascular function, acute illness (e.g., fever, dehydration, arrhythmia), mild cognitive impairment, new medication, and environmental change^7–15^. In addition, falls cause the injuries that require treatment^16^, and can result in serious complications^6,17,18^. For instance, traumatic cerebral hemorrhage cases due to fall accidents account for two-thirds of deaths, and serious injuries due to falls cause a loss of disability-adjusted life years^17–19^.

Falls are also a crucial issue in the case of inpatients, and risk factors for falls in hospitals are generally similar to those in the community. Risk factors of inpatients include older age, history of falls, cognitive impairment, dizziness, environmental hazards, impaired mobility, use of certain medications (e.g., influencing central nervous system or circulatory dynamics), and visual impairment, with a combination of these factors increasing the fall risk^20–23^.

Because the risk factors that cause falls are multifactorial and complicated, various guidelines related to inpatient falls are reported globally to address this problem. National Patient Safety Agency (NPSA)^24^, National Institute for Health and Care Excellence (NICE)^25^, and Robert Wood Johnson Foundation (RWJF)^26^ are representative guidelines. These guidelines recommend screening without a fall risk score to identify high-risk patients from the predictions of medical doctors, and conducting a multifactor fall risk assessment for each patient.

However, in Japan, many hospitals have traditionally used a score-based risk assessment that adds points depending on the number of risk factors of falls^27,28^; our hospital also uses score sheets. In the case of a scoring system, although clinical staff can easily evaluate score-risk assessment for falls, the results fall into uniform patterns for all patients, and the combination and correlation between clinical symptoms in individuals tends to be overlooked. Therefore, it is desirable that clinical staff comprehensively evaluate fall risk in each patient. Additionally, the fall risk assessment is mainly performed in Japan by nurses who conduct clinical observations at the bedside. Inpatient falls are defined in the National Database of Nursing Quality Indicators (NDNQI, 2010) as an “index that reflects the impact of nursing” and is indicated as a “nursing sensitive outcome”^29^. Furthermore, it has been reported that the length of time to care for patients and reduced allotment of registered nurses were associated with a high fall rate^30^. These results indicate that fall occurrence depends on the time that nurses spend on patient care. Clinical nurses need to be sensitive to changes in patient status and judge the risk of falls appropriately. They perform preventive interventions with assessment skills and clinical insights while maintaining clinical vigilance that is commensurate with medical standards^31^. Therefore, a fall risk assessment tool that enables nurses to identify the risk of falls appropriately and quickly in patients, and easily detect changes in patient’s risk is desirable.

In this study, we constructed an algorithm for a fall risk assessment which clinical staff including nurses can use to easily identify the essential risk factors for falls. It emphasizes the correlation and combination between essential risk factors for falls in hospitalized patients.

## Results

### Univariate analysis of each fall risk factor

We examined 36 fall risk factors between non-fall and fall groups. The results showed that the proportions of past history, muscle weakness of the lower limbs, need of transfer assistance, and night urination were > 70% in the fall group. Additionally, risk factors such as sex, visual impairment, dysesthesia, bedridden, chemotherapy, use of urethral catheter, hypoxemia, orthostatic hypotension, and hypoglycemia were not significant (Table 1).

**Table 1:** Characteristics of patients in the non-fall and fall groups of the training dataset.

	Non-fall (n = 56,673)	Fall (n = 238)	*p*-value
Age(year), median, IQR	67.0 (54.0, 75.0)	71.5 (63.0, 80.0)	< 0.001
Male, *n* (%)	29,887 (52.7)	139 (58.4)	0.09
Fall history, *n* (%)	14,551 (25.7)	169 (71.0)	< 0.001
Syncope, *n* (%)	4427 (7.8)	31 (13.0)	0.005
Visual impairment, *n* (%)	15,882 (28.0)	77 (32.4)	0.15
Auditory impairment, *n* (%)	7188 (12.7)	53 (22.3)	< 0.001
Paralysis, *n* (%)	2256 (4.0)	25 (10.5)	< 0.001
Dysesthesia, *n* (%)	11,776 (20.8)	59 (24.8)	0.13
Joint contracture/deformation, *n* (%)	569 (1.0)	7 (2.9)	0.01
Muscle weakness of the lower limbs, *n* (%)	25,391 (44.8)	203 (85.3)	< 0.001
Use of a walking aid, *n* (%)	16,216 (28.6)	153 (64.3)	< 0.001
Need of transfer assistance, *n* (%)	21,999 (38.8)	181 (76.1)	< 0.001
Stagger, *n* (%)	11,371 (20.1)	151 (63.4)	< 0.001
Bedridden status, *n* (%)	8167 (14.4)	45 (18.9)	0.05
Presence of infusion route/tube, *n* (%)	26,322 (46.4)	141 (59.2)	< 0.001
Impaired consciousness, *n* (%)	3069 (5.4)	36 (15.1)	< 0.001
Dementia, *n* (%)	4840 (8.5)	73 (30.7)	< 0.001
Impaired judgment/comprehension, *n* (%)	6708 (11.8)	104 (43.7)	< 0.001
Delirium, *n* (%)	889 (1.6)	28 (11.8)	< 0.001
Memory disturbance, *n* (%)	3434 (6.1)	55 (23.1)	< 0.001
Use of analgesic, *n* (%)	16,908 (29.8)	92 (38.7)	0.004
Use of opioid, *n* (%)	3810 (6.7)	27 (11.3)	0.009
Use of sleeping drug, *n* (%)	12,458 (22.0)	102 (42.9)	< 0.001
Use of anti-Parkinson’s disease agent, *n* (%)	419 (0.7)	9 (3.8)	< 0.001
Use of hypotensive diuretic, *n* (%)	10,956 (19.3)	65 (27.3)	0.003
Use of laxative, *n* (%)	13,933 (24.6)	90 (37.8)	< 0.001
Chemotherapy, *n* (%)	8078 (14.3)	33 (13.9)	0.93
Fecal/urinary incontinence, *n* (%)	4387 (7.7)	67 (28.2)	< 0.001
Pollakiuria, *n* (%)	3990 (7.0)	27 (11.3)	0.015
Need of toileting assistance, *n* (%)	14,458 (25.5)	146 (61.3)	< 0.001
Use of urethral catheter, *n* (%)	9227 (16.3)	37 (15.5)	0.86
Night urination, *n* (%)	39,077 (69.0)	192 (80.7)	< 0.001
Anemia (≤ Hb 9 mg/dl), *n* (%)	4051 (7.1)	37 (15.5)	< 0.001
Hypoxemia, *n* (%)	1528 (2.7)	9 (3.8)	0.31
Orthostatic hypotension, *n* (%)	913 (1.6)	4 (1.7)	0.80
Hypoglycemia, *n* (%)	534 (0.9)	3 (1.3)	0.50

*IQR* interquartile range.

### Correlation between fall and each fall risk factor

**Figure 1:**
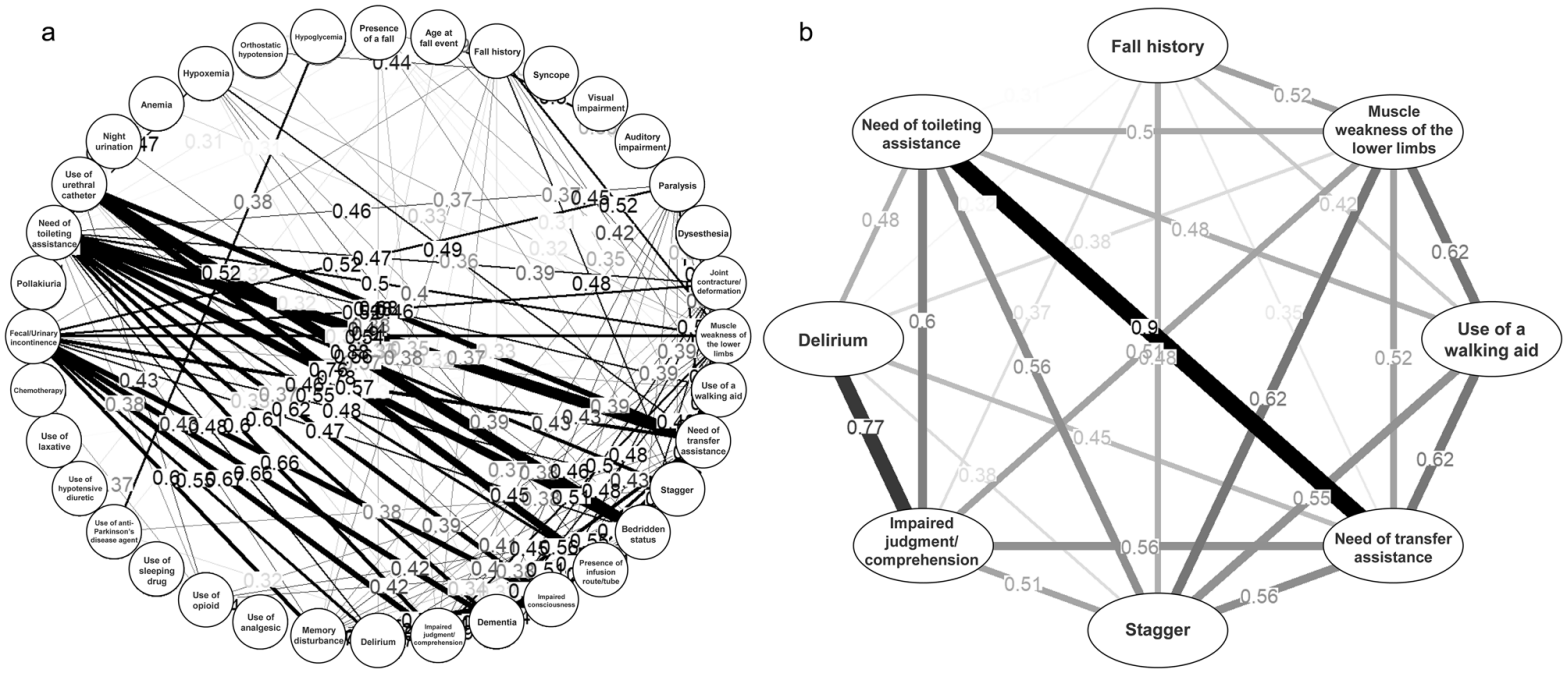
Correlation coefficients between fall risk factors with a fall event. The relationship between nominal variables and continuous variables or nominal variables were evaluated by polyserial or polychoric correlation coefficients, respectively. Correlation coefficients between fall risk factors are shown, including presence of a fall **(a)** and extracted risk factors that were related to presence of a fall **(b)**. To draw these figures, package qgraph (https://cran.r-project.org/web/packages/qgraph/qgraph.pdf) in R (version 4.0.2, URL: https://cran.r-project.org/bin/windows/base/old/4.0.2/) was utilized. In addition, we removed the abbreviations in outputted figures from qgraph and changed to non-abbreviated words. Correlation coefficients > 0.30 or < -0.30 are presented as plot of correlation. The line density shows the strength between each term (coefficient).

Polychoric and polyserial correlation coefficients are presented in Supplementary Table S1 online, and a plot of these correlations showing coefficients > 0.30 is presented (Fig. 1a). Fall history (0.39), muscle weakness of the lower limbs (0.37), use of a walking aid (0.31), need of transfer assistance (0.32), stagger (0.39), impaired judgment/comprehension (0.35), delirium (0.35), and need of toileting assistance (0.31) were related to the presence of a fall. Additionally, we focused on the relationship between the above fall event-related risk factors. A close relationship between each risk factor was confirmed (Fig. 1b).

### Investigation of important fall risk factors

**Figure 2:**
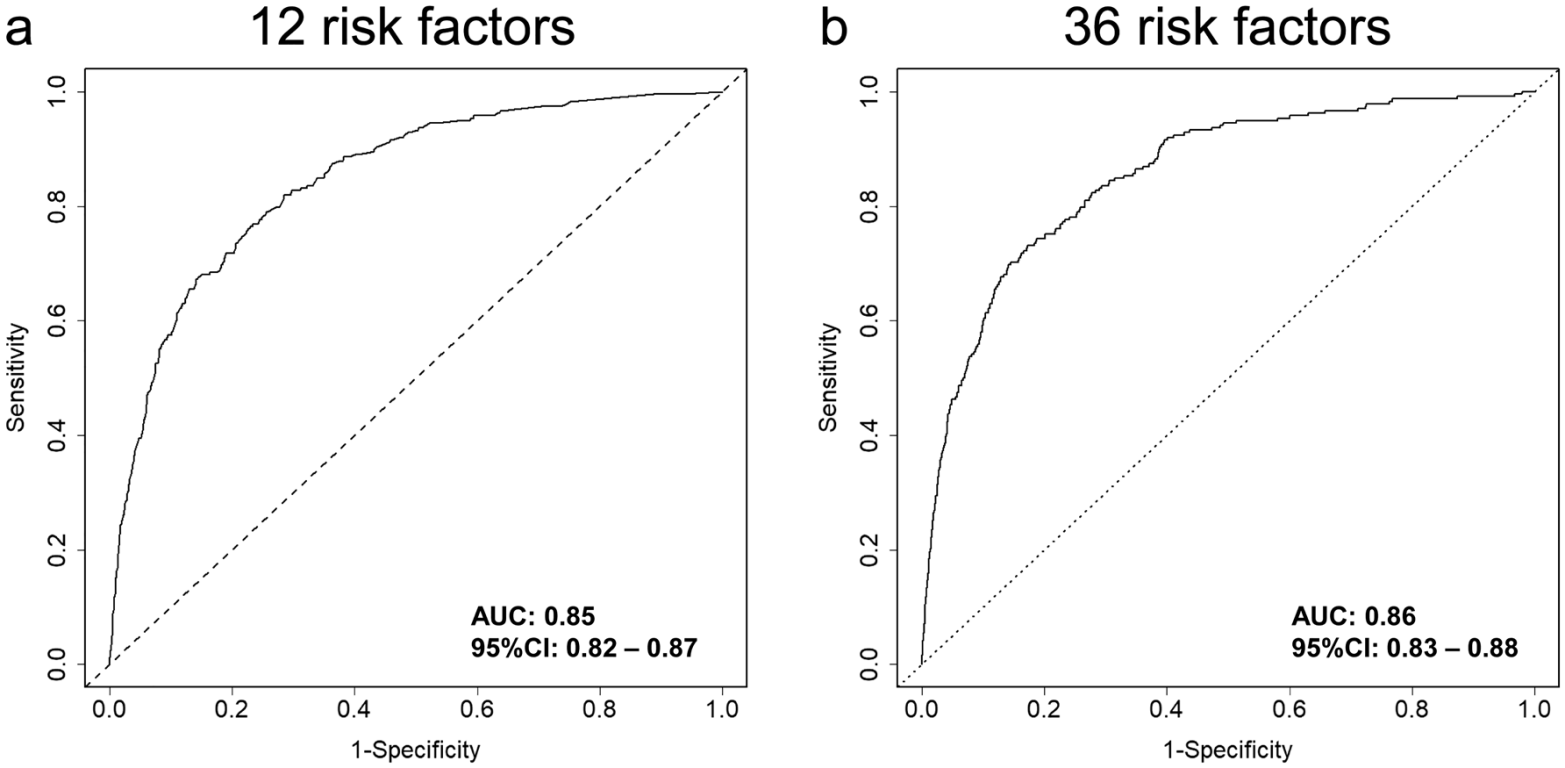
Receiver operating characteristic (ROC) curves. The predicted probabilities for a fall were estimated by two logistic models from 12 and 36 risk factors. ROC curves from predicted probabilities in 12 **(a)** and 36 **(b)** risk factors were plotted. To draw these figures, R (version 4.0.2, URL: https://cran.r-project.org/bin/windows/base/old/4.0.2/) was utilized.

We performed univariate logistic regression analysis using the presence or absence of falls as the objective variable and 36 fall risk factors as explanatory variables (Table 2). Next, multivariable logistic regression analysis with backward stepwise selection was performed without risk factors that were not significant in univariate logistic regression analysis (Table 3). Altogether, 12 important risk factors were extracted as follows: fall history (odds ratio [OR]: 2.99, 95% confidence interval [CI]: 2.20–4.07), muscle weakness of the lower limbs (OR: 1.96, 95%CI: 1.30–2.96), use of a walking aid (OR: 1.34, 95%CI: 0.98–1.82), stagger (OR: 2.00, 95%CI: 1.46–2.75), presence of infusion routes/tubes (OR: 1.42, 95%CI: 1.08–1.86), impaired judgment/comprehension (OR: 1.94, 95%CI: 1.41–2.67), impaired consciousness (OR: 0.58, 95%CI: 0.37–0.92), delirium (OR: 2.56, 95%CI: 1.56–4.18), use of sleeping drug (OR: 1.63, 95%CI: 1.25–2.12), urine or fecal incontinence (OR: 1.35, 95%CI: 0.97–1.87), need of toileting assistance (OR: 1.53, 95%CI: 1.11–2.10), and night urination (OR: 1.74, 95%CI: 1.24–2.44). Furthermore, the area under the receiver operating characteristic (ROC) curve (AUC), induced by predicted probability of the logistic model with 12 risk factors, was 0.85 (95%CI: 0.82–0.87), the AUC from 36 risk factors was 0.86 (95%CI: 0.83–0.88), and two ROC curves indicated similar results (Fig. 2). However, the predicted probability values from two logistic models were low (Supplementary Fig. S1 online), indicating that it may be difficult to predict falls in the actual clinical setting regardless of statistical analysis.

**Table 2:** Univariate logistic analysis for fall predictors.

Exogenous variable	OR	95%Cl	*p*-value
Age	1.03	(1.02, 1.04)	< 0.001
Male*	1.26	(0.97, 1.63)	0.08
Fall history*	7.09	(5.36, 9.30)	< 0.001
Syncope*	1.77	(1.21, 2.58)	0.003
Visual impairment*	1.23	(0.94, 1.61)	0.14
Auditory impairment*	1.97	(1.45, 2.68)	< 0.001
Paralysis*	2.83	(1.87, 4.29)	< 0.001
Dysesthesia*	1.26	(0.94, 1.69)	0.13
Joint contracture/deformation*	2.99	(1.40, 6.37)	0.005
Muscle weakness of the lower limbs*	7.15	(4.99, 10.23)	< 0.001
Use of a walking aid*	4.49	(3.44, 5.86)	< 0.001
Need of transfer assistance*	5.01	(3.71, 6.74)	< 0.001
Stagger*	6.91	(5.31, 9.01)	< 0.001
Bedridden status*	1.38	(1.00,1.92)	0.05
Presence of infusion route/tube*	1.68	(1.29, 2.17)	< 0.001
Impaired consciousness*	3.11	(2.18, 4.45)	< 0.001
Dementia*	4.74	(3.59, 6.25)	< 0.001
Impaired judgment/comprehension*	5.78	(4.47, 7.48)	< 0.001
Delirium*	8.37	(5.61, 12.48)	< 0.001
Memory disturbance*	4.66	(3.44, 6.31)	< 0.001
Use of analgesic*	1.48	(1.14, 1.92)	0.003
Use of opioid*	1.78	(1.19, 2.65)	0.005
Use of sleeping drug*	2.66	(2.06, 3.44)	< 0.001
Use of anti-Parkinson’s disease agent*	5.28	(2.69, 10.34)	< 0.001
Use of hypotensive diuretic*	1.57	(1.18, 2.09)	0.002
Use of laxative*	1.87	(1.43, 2.43)	< 0.001
Chemotherapy*	0.97	(0.67, 1.40)	0.86
Fecal/urinary incontinence*	4.67	(3.51, 6.20)	< 0.001
Pollakiuria*	1.69	(1.13, 2.53)	0.01
Need of toileting assistance*	4.63	(3.57, 6.02)	< 0.001
Use of urethral catheter*	0.95	(0.67, 1.35)	0.76
Night urination*	1.88	(1.36, 2.59)	< 0.001
Anemia (≤ Hb 9 mg/dl)*	2.39	(1.68, 3.40)	< 0.001
Hypoxemia*	1.42	(0.73, 2.77)	0.31
Orthostatic hypotension*	1.04	(0.39, 2.81)	0.93
Hypoglycemia*	1.34	(0.43, 4.20)	0.61

The decimal points of OR and 95%CI are rounded up to the third place and displayed to the second decimal place.

*OR* odds ratio, *CI* confidence interval.

*Negative (ref) positive.

**Table 3:** Multivariate logistic analysis for fall predictors.

Exogenous variable	OR	95%Cl	*p*-value
Fall history*	2.99	(2.20, 4.07)	< 0.001
Muscle weakness of the lower limbs*	1.96	(1.30, 2.96)	0..001
The use of a walking aid*	1.34	(0.98, 1.82)	0.07
Stagger*	2.00	(1.46, 2.75)	< 0.001
Presence of infusion route/tube*	1.42	(1.08, 1.86)	0.012
Impaired consciousness*	0.58	(0.37, 0.92)	0.020
Impaired judgment/comprehension*	1.94	(1.41, 2.67)	< 0.001
Delirium*	2.56	(1.56, 4.18)	< 0.001
Use of sleeping drug*	1.63	(1.25, 2.12)	< 0.001
Fecal/urinary incontinence*	1.35	(0.97, 1.87)	0.07
Need of toileting assistance*	1.53	(1.11, 2.10)	0.010
Night urination*	1.74	(1.24, 2.44)	0.001

The decimal points of OR and 95%CI are rounded up to the third place and displayed to the second decimal place.

*OR* odds ratio, *CI* confidence interval.

*Negative (ref) positive.

### Generation of an algorithm to assess fall risk

**Figure 3:**
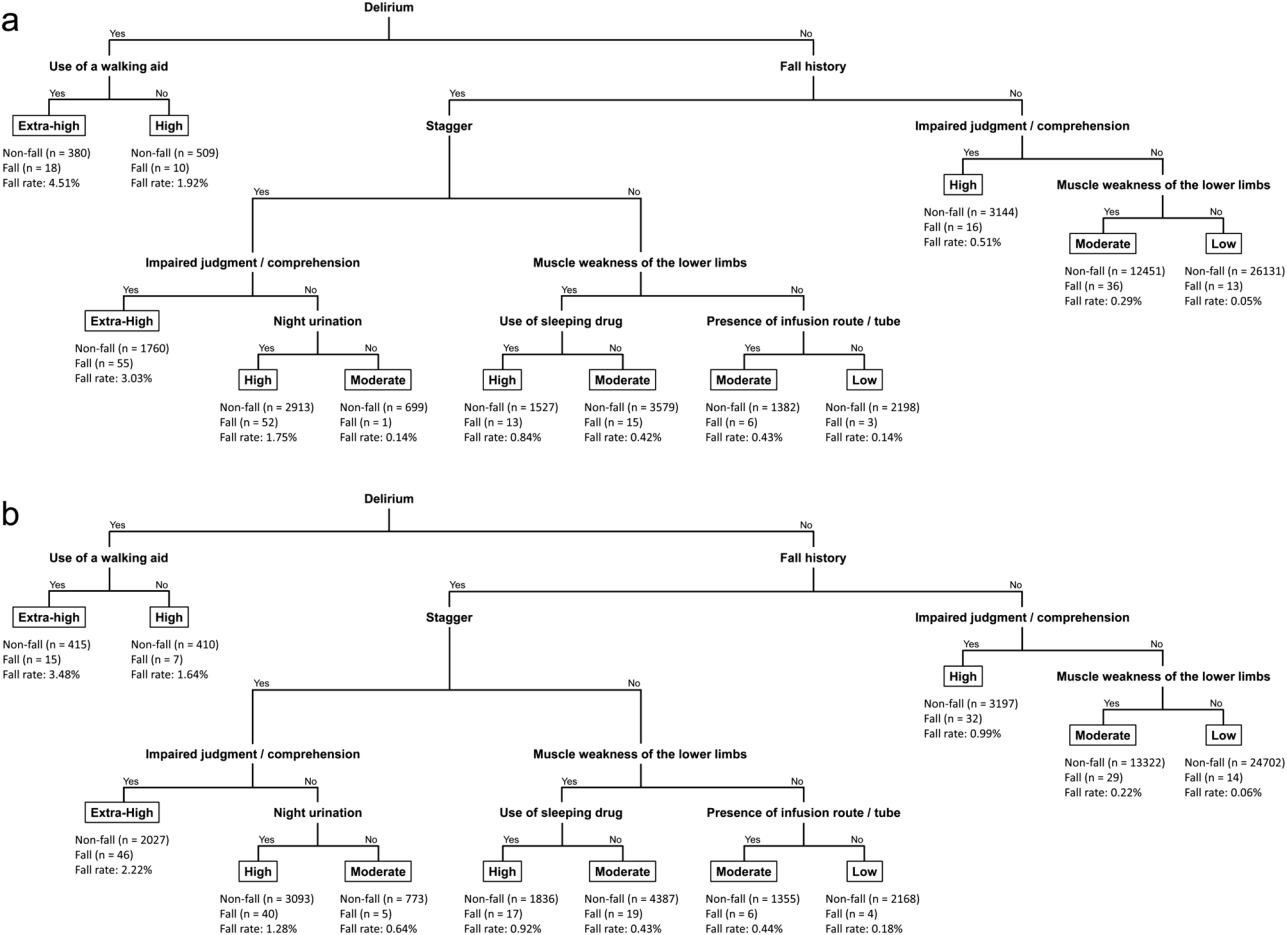
The algorithm for recognizing and estimating the risk level of falls. Training **(a)** and validation **(b)** datasets are presented. Risk level classified cases into low, moderate, high, and extra-high risk.

Next, we generated an algorithm for fall risk assessment that clinical staff including nurses can easily use to determine fall risk from a combination of factors including a patient’s history or clinical state. Therefore, decision tree analysis utilizing the above 12 important risk factors was performed. After discussions between a biostatistician and medical doctors and nurses, we generated an algorithm with nine essential risk factors: delirium, fall history, use of a walking aid, stagger, impaired judgment/comprehension, muscle weakness of the lower limbs, night urination, use of sleeping drug, and presence of infusion route/tube (Fig. 3a). Next, this algorithm conveniently classified cases into low, moderate, high, and extra-high risk by hierarchical combination of the patient’s information. Furthermore, we evaluated the algorithm using a validation set and confirmed its reliability (Fig. 3b).

## Discussion

Falls of inpatients can cause serious complications or prolonged hospitalization^32^. However, because inpatients naturally take care not to fall by themselves or with clinical staff, falls are rare events, and their accurate prediction is difficult. Actually, the predicted probability from our two logistic models showed low values. Therefore, there is a need for a fall risk assessment tool that clinical staff including nurses can use to appropriately and quickly identify essential fall risk for inpatients and easily detect changes in a patient’s risk. In this study, we evaluated 36 fall risk factors from inpatients, and created an algorithm for fall risk assessment that considers the correlation and combination between risk factors of falls in the actual clinical setting. This algorithm has four notable features.

First, we did not adopt a score-based risk assessment. Various fall risk screening tools have been previously proposed, with these tools mainly adopting score-based risk assessment that added points depending on the number of fall risk factors and set a cutoff value^33^. Scoring tools are particularly useful for acute hospitals because clinical staff need to judge the possibility of fall risk quickly. On the other hand, to reach a score in a timely manner, they may unconsciously fail to focus on clinical symptoms and combinations of factors that may cause a fall. Therefore, we generated an algorithm using clinical symptoms without converting them to a score. In this regard, nine fall risk factors used in the generated algorithm were reported as essential fall risk factors^21,24,34^, and our used risk factors are similar to those used for other screening tools^33^.

Our algorithm also considered the correlation between fall risk factors. When a patient fell, they usually had multiple fall risk factors that were not independent from each other. For instance, in our analysis, a patient with muscle weakness of the lower limbs tended to also show stagger or impaired judgment/comprehension, use a walking aid, and need transfer assistance. Accordingly, we hierarchically organized more important fall risk factors to focus on fall-positive cases. We set delirium as the first branch because clinical staff including nurses should intervene for delirium as a critical fall risk factor with need of treatment as early as possible^35–37^. Additionally, when delirium was positive, use of a walking aid was more important. Although there was moderate correlation between use of a walking aid and muscle weakness of the lower limbs, delirium was an emergency situation and clinical staff need to quickly respond to the state. Therefore, we choose the use of a walking aid that clinical staff can easily grasp. When delirium was negative, we set fall history as the second branch. According to a United States report, approximately 50% of individuals in a long-term care setting fall every year, and almost 60% of those with a falling history in the previous year will have a subsequent fall^38^. Additionally, the proportion of a past history of fall group in our research was over 70%, therefore we set fall history as the second branch. Next, we regarded stagger as serious in the cases with a fall history, and subsequently emphasized the combination of certain risk factors such as impaired judgement/comprehension, muscle weakness of the lower limbs, night urination, use of sleeping drug, and presence of infusion route/tube. Unlike past history, it is possible that these clinical signs are affected by surgical operation or medication during hospitalization, and the risk level for a fall fluctuated owing to the presence or absence of these factors. This tendency was fundamentally similar in the cases without delirium and a fall history. In these populations, the combination of impaired judgement/comprehension and muscle weakness of the lower limbs was considered as the foundation of the fall risk assessment. However, our algorithm did not include impaired consciousness, fecal/urinary incontinence, and need for toileting assistance, although they were among the 12 risk factors identified as important. The odds ratio for impaired consciousness for a fall incident was 0.58 in the multivariate logistic analysis and considering multiple fall risk factors, this sign was a marker for detecting the non-fall group rather than the fall group. Moreover, this sign was lower in the hierarchy in the decision tree analysis. Therefore, when this sign was added in our algorithm, it was possible that clinical staff, including nurses, could be confused as to whether the patient was likely to fall. Therefore, we excluded this sign from the algorithm. In addition, the decision tree analysis showed that fecal/urinary incontinence and need for toileting assistance were lower in the hierarchy and had lower importance for fall risk compared with the other nine risk factors. We discussed whether these two signs should be included in the algorithm from the perspective of the actual clinical setting. Based on our study concept of developing an algorithm that clinical staff including nurses, can use to easily identify essential risk factors for falls, we excluded these two factors to avoid staff needing to check many clinical symptoms.

Second, we conveniently classified the fall risk level into four groups (extra-high, high, moderate, and low) according to the risk of fall on the basis of clinical findings or inpatient status. As described above, although a fall risk assessment that considers the correlation and combination between risk factors is important, grasping of a change of risk level (e.g., administration of a sleeping drug to a case with a fall history and muscle weakness of the lower limbs) during hospitalization are needed. Therefore, after deciding on the branches of the algorithm, we discussed fall risk levels with reference to the fall rate in each node and the actual clinical setting. We defined cases where the fall rate was 2.00% or more as being at extra-high risk. A fall rate between 0.50 and 1.99% was defined as high risk. A fall rate between 0.20 and 0.49% was considered to reflect moderate risk, and a fall rate under 0.20% represented low risk. In this regard, when a patient had fall history and stagger without impaired judgment/comprehension and night urination, we defined that patient as having moderate risk despite the low fall rate in the training dataset, because fall history and stagger were important fall risk factors in the second and third branches. Using these criteria, we set four risk levels as a simple indicator.

Third, we generated the algorithm with assuming actual clinical setting to emphasize a problem-solving method. In Japan, fall assessment is mainly performed by nurses. They evaluate the fall risk for each inpatient, then provide preventive interventions according to individual cases. Namely, to avoid a fall and subsequent complications, they are demanded care processes by recognizing fall risks of inpatients and performing an assessment. In this case, although more information of fall risks there are, the more proper planning of care processes may be, excessive information gives great concern that loses sight of the essence in various risk factors. Additionally, confirming the presence or absence of numerous risk factors requires considerable time and the ability to respond promptly in fall risk assessment is decreased. Consequently, nurses may not be able to provide an appropriate care process for inpatients. Thus, we referred to certain guidelines namely algorithms of the American Academy of Geriatrics (AGS) and British Society of Geriatrics (BGS)^39–41^. They primally place importance on some of the essential fall risk factors, then assess risk comprehensively and perform a preventive intervention depending on the patient’s other conditions^39–41^. On the basis of these concepts, we also constructed an algorithm that nurses can use to identify essential fall risks appropriately and quickly from various fall risks. For instance, in our algorithm, because cases without delirium and a fall history had a low fall risk level in comparison to cases with delirium or a fall history, nurses preferentially evaluated the presence of impaired judgment/comprehension and muscle weakness of the lower limbs. Next, they performed a preventive intervention on judging the inpatient’s other conditions. This process presumes an actual clinical setting and is therefore a practical problem-solving method.

Fourth, using the clinical setting to emphasize the problem-solving method offered by our algorithm may help it to be accepted other clinical staff (e.g., medical doctors and pharmacists). Falls are of interest for other clinical staff as well as nurses^42,43^; these staff may feel that our algorithm is applicable to the evaluation of fall risk. Because our algorithm adopted clinical symptoms rather than a score system and offers a simple assessment of fall risk, information about fall risk for a patient can be shared with various clinical staff, including among nurses. Furthermore, our algorithm based on clinical symptoms allows clinical staff, such as medical doctors and pharmacists, to correlate a patient’s fall risk with their medical condition, treatment, and medication. As a result, all clinical staff may be able to contribute to preventive interventions for falls in a coordinated manner, which will improve these interventions.

Our algorithm was developed considering the above four points. In this regard, our research has some limitations. First, our study was performed in a single center. Although risk factors that we used in our algorithm have been reported as essential fall risk factors^21,24,34^, the extent to which our algorithm is applicable to general population of inpatients may elicit discussion. Second, our research is a retrospective study and the time of the fall within the fall history was not obvious. Therefore, to demonstrate the usefulness of this algorithm, a further prospective study is needed. Third, because inpatients are repeatedly evaluated for fall risk at transition points such as admission, start of medication, and operation, longitudinal information for essential fall risks per inpatient are increased. Consequently, the recognition of temporal changes in an inpatient’s condition may be scarcer compared with the current condition. However, by setting flags for essential risk factors in an electronic medical chart system, it is possible to perform retroactive confirmation, and understand the current situation based on past condition. Additionally, this flagged system is useful for consistent quality control of fall risk assessment. Fourth, although we extracted some essential fall risk factors to generate an algorithm, this algorithm is only the groundwork that nurses can the use to recognize these risk factors appropriately and quickly. Therefore, nurses are demanded care processes on considering other inpatient’s conditions based on essential risk factors and are needed to be application performance. In this regard, because there is a limit to the application performance per nurse, close cooperation between clinical staff, in particular nurses and medical doctors, is needed. Our algorithm can be considered convenient because various clinical staff can easily share patient information related to fall risk factors.

We have generated an algorithm that clinical staff including nurses can use to assess essential fall risks appropriately and quickly. Although further prospective study is needed, using this algorithm may help realize a more suitable fall risk management of inpatients.

## Methods

### Patients

Generally, reports of fall accidents that occur in a hospital are based on the subjectivity of the finders or reporters. However, in the clinical setting, it can be difficult to determine exactly at which point the patient fell. Therefore, in this study, falls were defined as events in which the patients unintentionally fell.

Clinical data were collected from fall risk assessment score sheets of the nursing information system and incident report system at Kumamoto University Hospital (Kumamoto, Japan) between October 2017 and September 2019. In our hospital, patients were repeatedly checked for fall risk by nurses because clinical symptoms changed by interventions such as medical treatments (e.g., medication, intravenous drip, operation). Consequently, a patient had varied clinical data on fall risk assessment at several time points. Therefore, in the case of patients without a fall during hospitalization (non-fall group), plural clinical data were extracted depending on the frequency of the fall risk assessment; these data were assumed as independent because of the changing condition. Conversely, for patients with a fall during hospitalization (fall group), only clinical data from the day that a fall occurred were used. This study was approved by the Institutional Review Board of Kumamoto University (Permit Number: 2028). Informed consent was obtained from all patients for participation in this study. In addition, when the patient was less than 18 years old, informed consent was obtained from the parent and/or legal guardian. All experiments were performed in accordance with the Declaration of Helsinki.

### Statistical analysis

**Figure 4:**
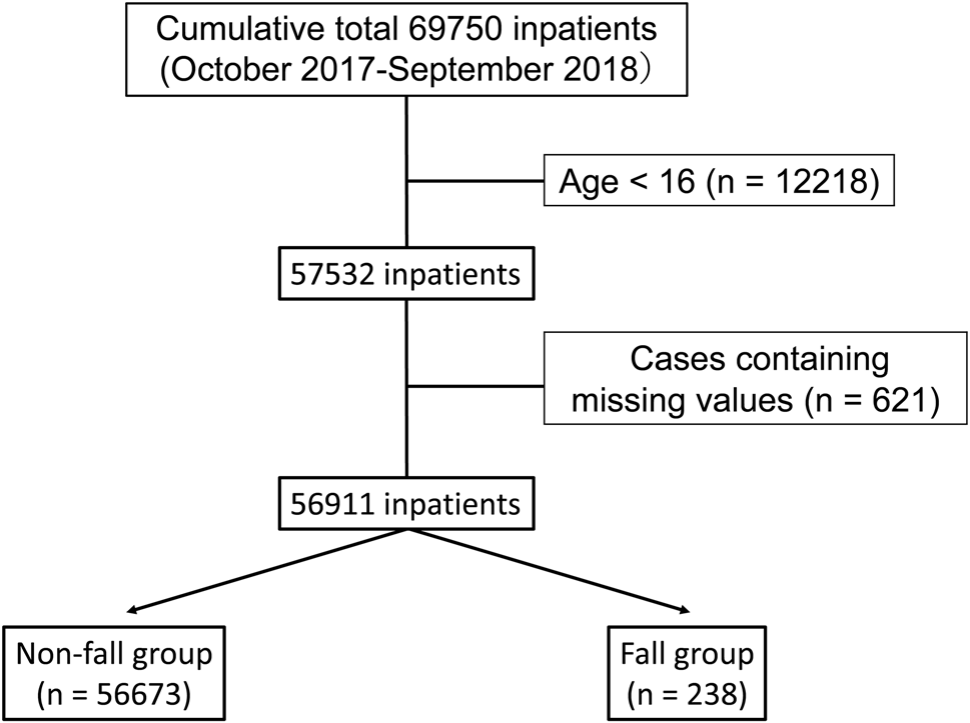
Overview of inpatient selection process for the training dataset.

A retrospective observational study was performed at Kumamoto University Hospital. Sample size was determined by consideration of the number of inpatients to Kumamoto University Hospital during the survey period. The dataset was divided into two subsets: one dataset was used for training and comprised 56,911 inpatients (non-fall, 56,673; fall, 238) hospitalized between October 2017 and September 2018 (Table 1); the other dataset was used for validation and comprised 57,929 inpatients (non-fall, 57,695; fall, 234) hospitalized between October 2018 and September 2019 (Supplementary Table S2 online). Exclusion criteria were patients who were < 16 years old and with missing values (Fig. 4). Under these conditions, the variables for analysis were collected from fall risk assessment score sheets as follows: age, sex, fall history, syncope, visual impairment, auditory impairment, paralysis, dysesthesia, joint contracture/deformation, muscle weakness of the lower limbs, use of a walking aid, need of transfer assistance, stagger, bedridden status, presence of infusion route/tube, impaired consciousness, dementia, impaired judgement/comprehension, delirium, memory disturbance, use of analgesic, use of opioid, use of sleeping drug, use of anti-Parkinson’s disease agent, use of hypotensive diuretic, use of laxative, chemotherapy, fecal/urinary incontinence, pollakiuria, need of toileting assistance, use of urethral catheter, night urination, anemia (≤ Hb 9 mg/dl), hypoxemia, orthostatic hypotension, and hypoglycemia.

In the training dataset, Welch’s *t*-test and Fisher’s exact test were performed for continuous and categorical variables, respectively. To evaluate the relationship between variables, polychoric and polyserial correlation coefficients were examined. Univariate logistic regression analyses using the above variables were performed to discriminate between the fall and non-fall groups. Additionally, a multiple logistic regression analysis was also used. In this model, covariates were selected by the backward stepwise Akaike Information Criteria method. Next, discrimination ability based on predicted probability of the logistic model was investigated using area under the ROC curve analyses. These analyses were performed using R version 4.0.2 (The R Foundation for Statistical Computing, Vienna, Austria, URL: https://cran.r-project.org/bin/windows/base/old/4.0.2/). In addition, Furthermore, we utilized decision tree analysis to identify important risk factors. JMP 14 (SAS Institute Inc., Cary, NC, USA) was used for this analysis. In JMP 14, when the objective variable (e.g., non-fall or fall) is categorical, it fits the probabilities estimated for the response levels, minimizing the residual log-likelihood chi-square^44^. Clinical validity of the above analyses was ascertained by discussing with nurses, medical doctors, and a biostatistician. Additionally, they were compared with risk factors previously reported in the literature. Consequently, we constructed an algorithm for fall risk assessment, and evaluated the algorithm using the validation dataset. The level of statistical significance was set at *P* < 0.05.

## Supplementary Information

Supplementary Information.

## Data Availability

The data supporting the findings of this study are available on request from T. Ikeda. The data are not publicly available as they contain information that could compromise the privacy of research participants.
