# Author Correction: Development of an algorithm for assessing fall risk in a Japanese inpatient population

**DOI:** 10.1038/s41598-021-98621-5

**Published:** 2021-09-17

**Authors:** Tomoko Nakanishi, Tokunori Ikeda, Taishi Nakamura, Yoshinori Yamanouchi, Akira Chikamoto, Koichiro Usuku

**Affiliations:** 1 Department of Medical Information Science, Graduate School of Medical Sciences, Kumamoto University, 1-1-1 Honjo, Chuou-ku, Kumamoto, 860-8556 Japan; 2 Department of Nursing, Kumamoto University Hospital, Kumamoto, Japan; 3 Department of Medical Information Sciences and Administration Planning, Kumamoto University Hospital, Kumamoto, Japan; 4 Laboratory of Clinical Pharmacology and Therapeutics, Faculty of Pharmaceutical Sciences, Sojo University, 4-22-1, Ikeda, Nishi-ku, Kumamoto, 860-8556 Japan; 5 Department of Medical Quality and Safety Management, Kumamoto University Hospital, Kumamoto, Japan

Correction to: *Scientific Reports* 10.1038/s41598-021-97483-1, published online 09 September 2021

The original version of this Article contained errors in the Affiliations.

Affiliation 1 was incorrectly given as ‘Department of Medical Information Sciences and Administration Planning, Graduate School of Medical Sciences, Kumamoto University Hospital, Kumamoto University, 1‑1‑1 Honjo, Kumamoto 860‑8556, Japan’. The correct affiliation is listed below:

Department of Medical Information Science, Graduate School of Medical Sciences, Kumamoto University, 1-1-1 Honjo, Chuou-ku, Kumamoto 860-8556, Japan

Additionally, Affiliation 4 was incorrectly given as ‘Laboratory of Clinical Pharmacology and Therapeutics, Faculty of Pharmaceutical Sciences, Sojo University, 4‑22‑1 Ikeda, Kumamoto 860‑0082, Japan’. The correct affiliation is listed below:

Laboratory of Clinical Pharmacology and Therapeutics, Faculty of Pharmaceutical Sciences, Sojo University, 4-22-1, Ikeda, Nishi-ku, Kumamoto 860-8556, Japan

The original Article and accompanying Supplementary Information file have been corrected.

