# Supplementary material for: Development of an algorithm for assessing fall risk in a Japanese inpatient population
Source: Sci Rep. 2021 Sep 9;11:17993. doi: 10.1038/s41598-021-97483-1 (PMC8429765; doi:10.1038/s41598-021-97483-1)
Supplement: Supplementary file 1 — Supplementary Information. [file 41598_2021_97483_MOESM1_ESM.pdf]

Supplementary Information

Development of an algorithm for assessing fall risk in a Japanese inpatient population

Tomoko Nakanishi[1,2*], Tokunori Ikeda[3,4*], Taishi Nakamura[1,3], Yoshinori Yamanouchi[1], Akira Chikamoto[5], Koichiro Usuku[1,3]

Author affiliations

[1]Department of Medical Information Science, Graduate School of Medical Sciences, Kumamoto University, 1-1-1 Honjo, Chuou-ku, Kumamoto 860-8556, Japan

[2]Department of Nursing, Kumamoto University Hospital, Kumamoto, Japan

[3]Department of Medical Information Sciences and Administration Planning, Kumamoto University Hospital, Kumamoto, Japan

[4]Laboratory of Clinical Pharmacology and Therapeutics, Faculty of Pharmaceutical Sciences, Sojo University, 4-22-1, Ikeda, Nishi-ku, Kumamoto 860-8556, Japan

[5]Department of Medical Quality and Safety Management, Kumamoto University Hospital, Kumamoto, Japan

***Corresponding authors**

Tomoko Nakanishi: t-nakanishi@kuh.kumamoto-u.ac.jp

Tokunori Ikeda: ryousei@ph.sojo-u.ac.jp

Supplementary Figure S1. Boxplot of predicted probabilities.

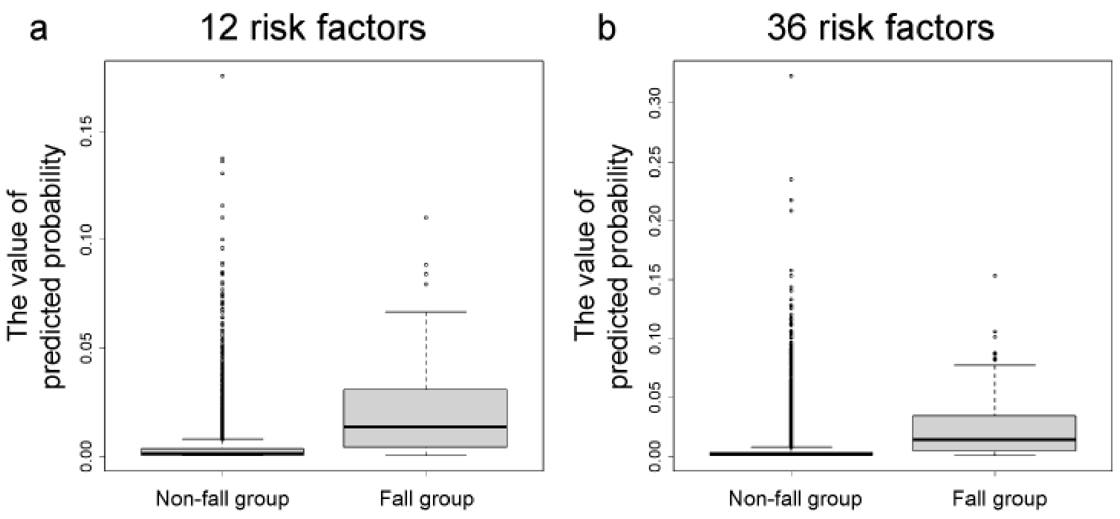

The predicted probabilities for a fall were estimated by two logistic models using 12 (a) and 36 (b) risk factors. To draw these figures, R (version 4.0.2, URL: https://cran.r-project.org/bin/windows/base/old/4.0.2/) was utilized.

Supplementary Table S1. Correlation coefficients between 36 risk factors.

	Presence of a fall	Age	Fall history	Syncope	Visual impairment	Auditory impairment	Paralysis	Dysesthesia	Joint contracture/deformation	Muscle weakness of the lower	Use of a walking aid	Need of transfer assistance	Stagger	Bedridden status	Presence of infusion route/tube	Impaired consciousness	Dementia	Impaired judgment/comprehension
Presence of a fall	1	0.15	0.39	0.1	0.04	0.13	0.18	0.04	0.15	0.37	0.31	0.32	0.39	0.07	0.11	0.2	0.3	0.35
Age	0.15	1	0.3	-0.06	0.15	0.5	0.02	0.11	0.03	0.45	0.3	0.21	0.2	0.02	0.01	0.25	0.21	0.27
Fall history	0.39	0.3	1	0.33	0.2	0.26	0.35	0.25	0.19	0.52	0.42	0.35	0.48	0.1	-0.03	0.28	0.33	0.37
Syncope	0.1	-0.06	0.33	1	0.04	0.04	0.07	0.09	0.07	0.13	0.09	0.13	0.22	0.08	0.02	0.16	0.18	0.19
Visual impairment	0.04	0.15	0.2	0.04	1	0.32	0.11	0.13	0.21	0.15	0.23	0.12	0.13	-0.06	-0.04	0.02	0.07	0.09
Auditory impairment	0.13	0.5	0.26	0.04	0.32	1	0.08	0.11	0.1	0.35	0.23	0.17	0.23	0.04	-0.01	0.12	0.15	0.21
Paralysis	0.18	0.02	0.35	0.07	0.11	0.08	1	0.15	0.55	0.47	0.4	0.5	0.39	0.39	0.09	0.39	0.43	0.43
Dysesthesia	0.04	0.11	0.25	0.09	0.13	0.11	0.15	1	0.12	0.33	0.24	0.13	0.22	0	-0.04	-0.09	-0.07	-0.05
Joint contracture/deformation	0.15	0.03	0.19	0.07	0.21	0.1	0.55	0.12	1	0.5	0.22	0.51	0.25	0.48	0.16	0.48	0.5	0.46
Muscle weakness of the lower limbs	0.37	0.45	0.52	0.13	0.15	0.35	0.47	0.33	0.5	1	0.62	0.52	0.62	0.24	0.15	0.43	0.48	0.51
Use of a walking aid	0.31	0.3	0.42	0.09	0.23	0.23	0.4	0.24	0.22	0.62	1	0.62	0.55	0.04	0.18	0.15	0.25	0.3
Need of transfer assistance	0.32	0.21	0.35	0.13	0.12	0.17	0.5	0.13	0.51	0.52	0.62	1	0.56	0.77	0.63	0.57	0.55	0.56
Stagger	0.39	0.2	0.48	0.22	0.13	0.23	0.39	0.22	0.25	0.62	0.55	0.56	1	0.26	0.17	0.39	0.47	0.51
Bedridden status	0.07	0.02	0.1	0.08	-0.06	0.04	0.39	0	0.48	0.24	0.04	0.77	0.26	1	0.71	0.59	0.51	0.47
Presence of infusion route/tube	0.11	0.01	-0.03	0.02	-0.04	-0.01	0.09	-0.04	0.16	0.15	0.18	0.63	0.17	0.71	1	0.29	0.23	0.22
Impaired consciousness	0.2	0.25	0.28	0.16	0.02	0.12	0.39	-0.09	0.48	0.43	0.15	0.57	0.39	0.59	0.29	1	0.84	0.84
Dementia	0.3	0.21	0.33	0.18	0.07	0.15	0.43	-0.07	0.5	0.48	0.25	0.55	0.47	0.51	0.23	0.84	1	0.99
Impaired judgment/comprehension	0.35	0.27	0.37	0.19	0.09	0.21	0.43	-0.05	0.46	0.51	0.3	0.56	0.51	0.47	0.22	0.84	0.99	1
Delirium	0.35	0.19	0.32	0.21	0.07	0.17	0.2	-0.05	0.38	0.38	0.17	0.45	0.38	0.32	0.09	0.81	0.75	0.77
Memory disturbance	0.28	0.33	0.38	0.17	0.12	0.25	0.39	-0.07	0.37	0.45	0.26	0.41	0.4	0.34	0.06	0.76	0.82	0.86
Use of analgesic	0.08	-0.04	0.07	-0.01	-0.11	0	0.01	0.19	-0.03	0.17	0.22	0.27	0.12	0.25	0.19	0.02	0.04	0.04
Use of opioid	0.1	-0.03	-0.05	-0.02	-0.1	-0.04	0	0.12	0.1	0.19	0.13	0.39	0.16	0.42	0.42	0.16	0.18	0.17
Use of sleeping drug	0.2	0.18	0.2	0.1	0.06	0.05	0.07	0.18	0.1	0.28	0.18	0.1	0.17	-0.03	0.01	0.1	0.19	0.2
Use of anti-Parkinson's agent	0.24	0.26	0.32	-0.03	0.07	0.1	0.14	0.04	0.28	0.28	0.26	0.24	0.38	0.08	-0.09	0.23	0.28	0.32
Use of hypotensive diuretic	0.09	0.32	0.14	0.02	0.08	0.13	0.16	0.06	0.11	0.24	0.18	0.14	0.14	0.02	-0.02	0.05	0.06	0.09
Use of laxative	0.13	0.13	0.21	0.09	0.03	0.08	0.23	0.22	0.3	0.3	0.19	0.14	0.21	0.04	0.03	0.1	0.13	0.15
Chemotherapy	0	-0.01	-0.06	0.02	-0.12	-0.08	-0.05	0.15	-0.14	0.01	-0.08	-0.19	-0.04	-0.18	0.15	-0.11	-0.05	-0.03
Fecal/Urinary incontinence	0.29	0.24	0.38	0.13	0.05	0.18	0.52	0.03	0.52	0.54	0.37	0.57	0.48	0.47	0.18	0.66	0.66	0.67
Pollakiuria	0.09	0.19	0.2	0.09	0.05	0.18	0.17	0.02	0.13	0.23	0.18	0.13	0.25	-0.02	-0.05	0.12	0.14	0.18
Need of toileting assistance	0.31	0.18	0.31	0.14	0.03	0.15	0.46	0.09	0.47	0.5	0.48	0.9	0.56	0.78	0.55	0.62	0.61	0.6
Use of urethral catheter	-0.01	0.05	0.01	0.03	-0.1	0.03	0.24	-0.05	0.26	0.16	0	0.68	0.17	0.88	0.76	0.46	0.37	0.33
Night urination	0.12	0.24	0.14	0.01	0.08	0.14	-0.22	0.08	-0.29	0.09	0.1	-0.22	0.07	-0.44	-0.17	-0.3	-0.2	-0.14
Anemia	0.16	0.02	0.14	0.11	0.01	0.06	0.09	0.08	0.23	0.33	0.2	0.27	0.29	0.2	0.27	0.2	0.23	0.22
Hypoxemia	0.05	0.1	0.04	0.15	0.02	0.09	-0.03	0.03	0.3	0.37	0.28	0.49	0.26	0.4	0.46	0.38	0.32	0.3
Orthostatic hypotension	0.01	-0.07	0.27	0.44	0.09	0.04	0.09	0.12	0.25	0.29	0.2	0.2	0.36	0.08	0.05	0.13	0.14	0.14
Hypoglycemia	0.04	-0.05	0.19	0.13	0.14	-0.04	0.07	0.24	0.11	0.17	0.12	0.06	0.12	-0.06	-0.07	-0.01	0	-0.01

	Delirium	Memory disturbance	Use of analgesic	Use of opioid	Use of sleeping drug	Use of anti-Parkinson's agent	Use of hypotensive diuretic	Use of laxative	Chemotherapy	Fecal/Urinary incontinence	Pollakiuria	Need of toileting assistance	Use of urethral catheter	Night urination	Anemia	Hypoxemia	Orthostatic hypotension	Hypoglycemia
Presence of a fall	0.35	0.28	0.08	0.1	0.2	0.24	0.09	0.13	0	0.29	0.09	0.31	-0.01	0.12	0.16	0.05	0.01	0.04
Age	0.19	0.33	-0.04	-0.03	0.18	0.26	0.32	0.13	-0.01	0.24	0.19	0.18	0.05	0.24	0.02	0.1	-0.07	-0.05
Fall history	0.32	0.38	0.07	-0.05	0.2	0.32	0.14	0.21	-0.06	0.38	0.2	0.31	0.01	0.14	0.14	0.04	0.27	0.19
Syncope	0.21	0.17	-0.01	-0.02	0.1	-0.03	0.02	0.09	0.02	0.13	0.09	0.14	0.03	0.01	0.11	0.15	0.44	0.13
Visual impairment	0.07	0.12	-0.11	-0.1	0.06	0.07	0.08	0.03	-0.12	0.05	0.05	0.03	-0.1	0.08	0.01	0.02	0.09	0.14
Auditory impairment	0.17	0.25	0	-0.04	0.05	0.1	0.13	0.08	-0.08	0.18	0.18	0.15	0.03	0.14	0.06	0.09	0.04	-0.04
Paralysis	0.2	0.39	0.01	0	0.07	0.14	0.16	0.23	-0.05	0.52	0.17	0.46	0.24	-0.22	0.09	-0.03	0.09	0.07
Dysesthesia	-0.05	-0.07	0.19	0.12	0.18	0.04	0.06	0.22	0.15	0.03	0.02	0.09	-0.05	0.08	0.08	0.03	0.12	0.24
Joint contracture/deformation	0.38	0.37	-0.03	0.1	0.1	0.28	0.11	0.3	-0.14	0.52	0.13	0.47	0.26	-0.29	0.23	0.3	0.25	0.11
Muscle weakness of the lower limbs	0.38	0.45	0.17	0.19	0.28	0.28	0.24	0.3	0.01	0.54	0.23	0.5	0.16	0.09	0.33	0.37	0.29	0.17
Use of a walking aid	0.17	0.26	0.22	0.13	0.18	0.26	0.18	0.19	-0.08	0.37	0.18	0.48	0	0.1	0.2	0.28	0.2	0.12
Need of transfer assistance	0.45	0.41	0.27	0.39	0.1	0.24	0.14	0.14	-0.19	0.57	0.13	0.9	0.68	-0.22	0.27	0.49	0.2	0.06
Stagger	0.38	0.4	0.12	0.16	0.17	0.38	0.14	0.21	-0.04	0.48	0.25	0.56	0.17	0.07	0.29	0.26	0.36	0.12
Bedridden status	0.32	0.34	0.25	0.42	-0.03	0.08	0.02	0.04	-0.18	0.47	-0.02	0.78	0.88	-0.44	0.2	0.4	0.08	-0.06
Presence of infusion route/tube	0.09	0.06	0.19	0.42	0.01	-0.09	-0.02	0.03	0.15	0.18	-0.05	0.55	0.76	-0.17	0.27	0.46	0.05	-0.07
Impaired consciousness	0.81	0.76	0.02	0.16	0.1	0.23	0.05	0.1	-0.11	0.66	0.12	0.62	0.46	-0.3	0.2	0.38	0.13	-0.01
Dementia	0.75	0.82	0.04	0.18	0.19	0.28	0.06	0.13	-0.05	0.66	0.14	0.61	0.37	-0.2	0.23	0.32	0.14	0
Impaired judgment/comprehension	0.77	0.86	0.04	0.17	0.2	0.32	0.09	0.15	-0.03	0.67	0.18	0.6	0.33	-0.14	0.22	0.3	0.14	-0.01
Delirium	1	0.67	0.05	0.09	0.27	0.29	-0.05	0.14	-0.06	0.55	0.22	0.48	0.19	-0.08	0.14	0.25	0.24	0.11
Memory disturbance	0.67	1	-0.06	-0.03	0.15	0.27	0.08	0.14	-0.09	0.6	0.2	0.43	0.16	-0.05	0.07	0.25	-0.06	-0.02
Use of analgesic	0.05	-0.06	1	0.54	0.14	-0.02	-0.03	0.08	0	0.04	-0.01	0.28	0.27	-0.15	0.13	0.09	0.05	0.02
Use of opioid	0.09	-0.03	0.54	1	0.04	-0.11	-0.15	0.19	0.37	0.06	-0.1	0.38	0.43	-0.13	0.27	0.19	0.08	-0.06
Use of sleeping drug	0.27	0.15	0.14	0.04	1	0.23	0.11	0.27	0.06	0.1	0.06	0.07	-0.06	0.11	0.13	0.2	0.12	-0.07
Use of anti-Parkinson's agent	0.29	0.27	-0.02	-0.11	0.23	1	-0.03	0.19	-0.24	0.29	0.29	0.24	0.05	0.06	-0.14	-0.05	0.1	-0.52
Use of hypotensive diuretic	-0.05	0.08	-0.03	-0.15	0.11	-0.03	1	0.08	-0.12	0.09	0.14	0.11	0.04	0.1	0.05	0.27	-0.08	0.1
Use of laxative	0.14	0.14	0.08	0.19	0.27	0.19	0.08	1	0.27	0.24	0.13	0.12	-0.02	0.1	0.16	0.15	0.18	0.06
Chemotherapy	-0.06	-0.09	0	0.37	0.06	-0.24	-0.12	0.27	1	-0.08	-0.06	-0.21	-0.18	0.23	0.23	-0.01	0.11	-0.13
Fecal/Urinary incontinence	0.55	0.6	0.04	0.06	0.1	0.29	0.09	0.24	-0.08	1	0.29	0.61	0.31	-0.19	0.24	0.28	0.16	0.09
Pollakiuria	0.22	0.2	-0.01	-0.1	0.06	0.29	0.14	0.13	-0.06	0.29	1	0.16	-0.02	0.28	0.05	-0.03	0.11	0.05
Need of toileting assistance	0.48	0.43	0.28	0.38	0.07	0.24	0.11	0.12	-0.21	0.61	0.16	1	0.7	-0.31	0.3	0.47	0.22	0.03
Use of urethral catheter	0.19	0.16	0.27	0.43	-0.06	0.05	0.04	-0.02	-0.18	0.31	-0.02	0.7	1	-0.45	0.17	0.32	0	-0.14
Night urination	-0.08	-0.05	-0.15	-0.13	0.11	0.06	0.1	0.1	0.23	-0.19	0.28	-0.31	-0.45	1	-0.01	-0.1	0.05	0.15
Anemia	0.14	0.07	0.13	0.27	0.13	-0.14	0.05	0.16	0.23	0.24	0.05	0.3	0.17	-0.01	1	0.26	0.15	-0.02
Hypoxemia	0.25	0.25	0.09	0.19	0.2	-0.05	0.27	0.15	-0.01	0.28	-0.03	0.47	0.32	-0.1	0.26	1	0.22	0
Orthostatic hypotension	0.24	-0.06	0.05	0.08	0.12	0.1	-0.08	0.18	0.11	0.16	0.11	0.22	0	0.05	0.15	0.22	1	0.31
Hypoglycemia	0.11	-0.02	0.02	-0.06	-0.07	-0.52	0.1	0.06	-0.13	0.09	0.05	0.03	-0.14	0.15	-0.02	0	0.31	1

The values of correlation coefficients are rounded up to the third place and displayed to the second decimal place.

Supplementary Table S2. Characteristics of patients with fall and non-fall group in validation dataset.

	Non-fall (n=57,695)	Fall (n=234)	*p*-Value
Age(year), median, IQR	67.0 (55.0, 75.0)	71.0 (63.0, 76.0)	<0.001
Male, *n* (%)	29433 (51.0)	127 (54.3)	0.33
Fall history, *n* (%)	16095 (27.9)	155 (66.2)	<0.001
Syncope, *n* (%)	4415 (7.7)	40 (17.1)	<0.001
Visual impairment, *n* (%)	16572 (28.7)	84 (35.9)	0.02
Auditory impairment, *n* (%)	7189 (12.5)	39 (16.7)	0.06
Paralysis, *n* (%)	2644 (4.8)	26 (11.1)	<0.001
Dysesthesia, *n* (%)	12165 (21.1)	68 (29.1)	0.004
Joint contracture/deformation, *n* (%)	577 (1.0)	7 (3.0)	0.01
Muscle weakness of the lower limbs, *n* (%)	27937 (48.4)	198 (84.6)	<0.001
Use of a walking aid, *n* (%)	16728 (29.0)	136 (58.1)	<0.001
Need of transfer assistance, *n* (%)	21930 (38.0)	165 (70.5)	<0.001
Stagger, *n* (%)	11941 (20.7)	143 (61.1)	<0.001
Bedridden status, *n* (%)	8263 (14.3)	41 (17.5)	0.16
Presence of infusion route/tube, *n* (%)	26971 (46.7)	134 (57.3)	0.002
Impaired consciousness, *n* (%)	3089 (5.4)	47 (20.1)	<0.001
Dementia, *n* (%)	5167 (9.0)	83 (35.5)	<0.001
Impaired judgment/comprehension, *n* (%)	7381(12.8)	113 (48.3)	<0.001
Delirium, *n* (%)	835 (1.4)	22 (9.4)	<0.001
Memory disturbance, *n* (%)	3518 (6.1)	42 (17.9)	<0.001
Use of analgesic, *n* (%)	18035 (31.3)	100 (42.7)	<0.001
Use of opioid, *n* (%)	3519 (6.1)	32 (13.7)	<0.001
Use of sleeping drug, *n* (%)	12883 (22.3)	95 (40.9)	<0.001
Use of anti-Parkinson's disease agent, *n* (%)	282 (0.5)	2 (0.9)	0.32
Use of hypotensive diuretic, *n* (%)	10703 (18.6)	61 (26.1)	0.005
Use of laxative, *n* (%)	14565 (25.3)	99 (42.3)	<0.001
Chemotherapy, *n* (%)	8979 (15.6)	47 (20.1)	0.07
Fecal/Urinary incontinence, *n* (%)	4263 (7.4)	55 (23.5)	<0.001
Pollakiuria, *n* (%)	3469 (6.0)	23 (9.8)	0.02
Need of toileting assistance, *n* (%)	14270 (24.7)	126 (53.8)	<0.001
Use of urethral catheter, *n* (%)	8977 (15.6)	27 (11.5)	0.1
Night urination, *n* (%)	40780 (70.7)	186 (79.5)	0.003
Anemia (≤ Hb 9mg/dl), *n* (%)	3950 (6.8)	29 (12.4)	0.003
Hypoxemia, *n* (%)	1476 (2.6)	9 (3.8)	0.21
Orthostatic hypotension, *n* (%)	927 (1.6)	16 (6.8)	<0.001
Hypoglycemia, *n* (%)	677 (1.2)	8 (3.4)	0.007

Abbreviations: IQR = interquartile range.
